# Shrinkage Study and Strength Aspects of Concrete with Foundry Sand and Coconut Shell as a Partial Replacement for Coarse and Fine Aggregate

**DOI:** 10.3390/ma14237420

**Published:** 2021-12-03

**Authors:** Kalyana Chakravarthy Polichetty Raja, Ilango Thaniarasu, Mohamed Abdelghany Elkotb, Khalid Ansari, C Ahamed Saleel

**Affiliations:** 1 Department of Civil Engineering, Vels Institute of Science, Technology & Advanced Studies, Chennai 600117, India; ilango.se@velsuniv.ac.in; 2 Mechanical Engineering Department, College of Engineering, King Khalid University, P.O. Box 394, Abha 61421, Saudi Arabia; melkotb@kku.edu.sa (M.A.E.); ahamedsaleel@gmail.com (C.A.S.); 3 Mechanical Engineering Department, Faculty of Engineering, Kafrelsheikh University, Sakha Road, Kafr Elsheikh 33516, Egypt; 4 Department of Civil Engineering, Yeshwantrao Chavan College of Engineeeing, Nagpur 441110, India; khalidshamim86@rediffmail.com

**Keywords:** concrete, waste foundry sand, coconut shell, shrinkage

## Abstract

The demand for natural aggregates (river sand) is increasing day by day, leading to the destruction of the environment, a burden that will be passed on to young people. Further, wastes from various industries are being dumped in landfills, which poses serious environmental problems. In order to ensure sustainability, both the issues mentioned above can be solved by utilizing industrial waste as aggregate replacement in the concrete construction industry. This research is done to find out the results using two substances viz., waste foundry sand (WFS) and coconut shell (CS) substitute for river sand and coarse aggregate. Many researchers have found the maximum benefits of substituted substances used in cement, which has material consistency. This current observation explores these strong waste properties of waste-infused concrete and cement, which experience shrinkage from drying out. The replacement levels for waste foundry sand were varied, between 10%, 20%, and 30%, and for CS, it was 10% and 20%. The experimental outcomes are evident for the strength, which increases by using WFS, whereas the strength decreases by increasing the CS level. The concrete that experiences shrinkage from drying out is included in the waste material, showing a higher magnitude of drying shrinkage than conventional concrete.

## 1. Introduction

Concrete is a continuously evolving material used to fulfill the projected civil infrastructure requirements of the 21st century. Sustainability and durability are the desirable characteristics of concrete infrastructure. There is an increasing concern about the use of natural resources and river sand mining for concrete production. The depletion of natural resources for civil infrastructure construction should be reduced and given high precedence by infrastructure designers. Adaption of sustainable construction materials along with durability are grand challenges for current and future civil engineers. Over the past few decades, researchers have experimented with different wastages obtained from industries such as foundry sand, steel slag, copper slag, palm oil clinker, etc., for river sand used as a substitute for concrete. More research was done on the first and third material out of the wastages mentioned above. Foundry sand, a by-product from the metal casting industry, is generated in large quantities and dumped as an environmentally degrading landfill. Foundry sand is more suitable for the replacement of fine aggregate when compared to other industrial wastes, which are fresh as well as hardened substances of cement. Khatib has widely studied this effect [1]; Coconut shell is discarded as agricultural waste, commonly dumped as landfill. Few researchers have studied the effect of utilizing coconut shells for non-structural applications. This effect has been widely studied by Pennarasi, G [2]; The alternate uses of coconut shells and their feasibility for structural applications were not fully evaluated.

The tremendous growth in the industrial sector has led to industrial waste products in a large quantity. Industrial growth faces a few challenges such as resource efficiency and productivity to ensure that the material is utilized effectively at all stages of its lifecycle till its disposal. Industrial waste is a material that is declared useless during any industrial activity such as production, mining, and construction. Based on the characteristics of the waste, it can be either classified as hazardous waste and non-hazardous waste. The disposal method depends on the type of waste generated. The most commonly adopted waste disposal methods are landfill and incineration. Almost all the countries face a problem of waste accumulation, and both of the disposal methods mentioned above cause high degradation to the environment. The incineration method causes air pollution due to the dispersion of very fine particles in the atmosphere, whereas the method of a landfill poses a serious impact on the environment through the leaching of toxic chemicals. We need to preserve the environment for our future generations through sustainable development [2]. Environment sustainability and circular economy have become a part of the industrial sector to reduce natural resources and minimize waste generation. The different types of waste generated from the industrial and agricultural sectors need to be categorized, and viable alternatives need to be studied. Globally, 11 billion tons of industrial waste were generated, and 600 million tons of agricultural waste is generated in India [3]. Industrial waste includes by-products such as slag, fly ash, sludge, glass, foundry sand, scrap metal, tires, etc. The agricultural waste produced as a result of various agricultural activities includes rice husk ash, bagasse, saw dust, groundnut shell, coconut shell, rice, and wheat straw. Numerous researches have been carried out to check the suitability of the above stated industrial/agricultural waste as a building material.

## 2. Literature Review

### 2.1. General

The author assessed that these concrete properties are incorporated with green foundry sand and chemical foundry sand. They came to the conclusion that the concretes which are made with metallurgical byproducts diffuse lower gravitational attraction as well as obtain maximum compressive strength at high temperature than ordinary concrete [4]. They examined concrete durability as well as abrasion resistance substances, which are incorporated with waste foundry sand in ratios of 0%, to *n*% which are a multiple of 5. Adding wastages generated from foundry industries enhanced the concrete strength. The concrete compressive strength increased to 8.25–17%, the modulus of elasticity by 1.67–6.35% and the split tensile strength to 3.55–10.40%, depending on the WFS content [5].

The replacement of 100% coarse aggregate with coconut shell and replaced cement with different percentages of ground granulated blast furnace slag (GGBS). The results revealed that the use of coconut shell for the entire replacement of coarse aggregate produces lightweight concrete, which ultimately reduces the compressive strength but achieves the target strength. The incorporation of ground granulated blast furnace slag (GGBS) for cement replacement increases the quality of concrete, since silica and alumina contents are used for the hydration process [5]. They examined the results that were achieved by substituting the coarse aggregate with 10%, 20%, 30%, and 40% of CS. The authors concluded that rising CS level in concrete decreases the compressive strength, but the strength can be maintained similar to conventional concrete either by reducing the percentage of weight of water and cement or by increasing the concrete content. The concrete flexural behavior with coconut shell, GGBS, and manufactured sand. The flexural behavior of under- and over-reinforced members with coconut shell were designed and tested for deflection and crack width. The results obtained are comparable with the permissible values stated by (IS:456 (2000). They characterized the plastic shrinkage and deflection behavior of coconut shell concrete through casting two-way slabs of size 533 mm × 838 mm × 40 mm. Five different concrete mixes with varying percentage (25%, 50%, 75%, 100%) of coconut shell were used as the coarse aggregate replacement [6]. The slab was tested on a loading frame that was supported on all four sides. The plastic shrinkage of slabs was measured in terms of the number of cracks formed, the maximum crack width, and crack length. The increase in percentage of CS decreases the compressive strength and the plastic shrinkage crack area, whereas the deflection increases. Though the deflection of CS concrete is higher than conventional concrete, it satisfies the maximum deflection requirement as per IS 456.The comparative study of concrete with coconut shell (CS) and palm kern shell (PKS) as coarse aggregates. CS and PKS are replaced in gradation of 0%, 25%, 50%, 75%, and 100% for two mix ratios such as (1:1:2 and 1:2:4). A total of 320 cubes were cast of size 100 × 100 × 100 mm to test the compressive strength of concrete. From the results, the authors have declared that the compressive strength of the concrete for all two mix ratios decreased with an increase in the percentage of the shells. However, the CS concrete showed higher compressive strength when compared to PKS concrete. Based on the comparative cost analysis, CS and PKS concrete have 30% and 42% cost reduction, respectively. It was decided that CS is more suitable than PKS for the replacement of conventional coarse aggregate on the basis of strength/economy ratio. From the extensive literature review conducted, it is evident that cement mixed with high quality silica sand which is substituted for fine aggregate and coconut shell as coarse aggregate needs to be experimented for shrinkage effects along with the strength properties to make it a suitable material for structural concrete [7].

### 2.2. Research Significance

The best way to utilize this industrial waste is to hide it inside the concrete without being detrimental to the environment. Although prior investigations have reported that the strength of concrete depends on high quality silica sand and coarse aggregate, the underlying mechanism of shrinkage was not fully understood. The factors affecting the shrinkage of concrete is due to concrete constituents, mixed proportion, age of concrete at loading, duration of loading, size of member, and environmental conditions (ACI Committee 209 (2005)). Drying shrinkage occurs when the volume changes due to loss of moisture content from the surface of the pores during the drying process of concrete. The code has also proposed standard values for shrinkage strain to be used in the design for normal ingredients of concrete such as river sand, crushed stone, ordinary Portland cement, and portable water. However, if the constituents of the concrete such as river sand, crushed stone, ordinary Portland cement, and portable water are being altered for sustainability, then it is necessary to predict the shrinkage of sustainable concrete. This study has examined the time dependent deformation of concrete, i.e., shrinkage by replacing fine aggregate with different percentages of discarded foundry sand from metal casting industries and by replacing coarse aggregate with different percentages of waste coconut shell from the agricultural industry. However, for validating the efficacy of sand and coconut shell to impart desirable quality criteria, standard tests were taken up to study the strength and durability standards. Attention given to the experiment is two-fold:

- A proportionate mix ratio for M20 grade of concrete is used to ascertain the strength, with different percentages of discarded foundry sand for natural river sand and coconut shell for coarse aggregate;
- To evaluate the concrete shrinkage behavior with various ratios of foundry sand and coconut shell as per ASTM standards.

## 3. Experimental Investigation

### 3.1. Materials

The mix-proportioning of M20 grade of concrete is made by evaluating the physical properties of concrete constituents such as cement, river sand, coarse aggregate foundry sand, and coconut shell, as per Indian Standards (IS:2386 (1963); IS:383 (1970)). A total of seven mix proportions including normal concrete, designated as CC, were designed as per IS: 10262 (2009) by considering a concrete and water proportion of 0.5. The foundry sand acts partially as a replacement for fine aggregate, with various percentages of replacement such as 10%, 20%, and 30% weight of the fine aggregate, and CS was used for substituting crushed stone by 10% and 20%. Mixes were combined and are depicted in Table 1.

The whole elements could be combined by heat as well as the aggregates added, which were in a saturated dry condition. As the water absorption is not same for different aggregates, correction for the water cement ratio was made and superplasticizer (CONPLAST SP430) was added (1% by weight of cement) for compensation. The mixing was done to achieve a uniform mixture. The specimens required for testing the mechanical properties were cast as per IS 1199 (1959)].

### 3.2. Methods

The fresh properties and hardened properties such as slump, compression strength, split tensile strength and flexural strength were found as per IS 516:1959. The experimentation was undergone for the first phase, in which coconut shell was partially replaced for coarse aggregate. The test was then conducted for fresh concrete and hardened concrete. The optimum percentage of replacement was derived. For fresh concrete, a slump test was conducted for various replacement. The tests conducted for hardened concrete are compressive strength, split tensile strength, flexural strength, and modulus of elasticity after 28 days of curing. The following testing methods are explained in detail.

#### 3.2.1. Slump Cone Test

**Figure 1:**
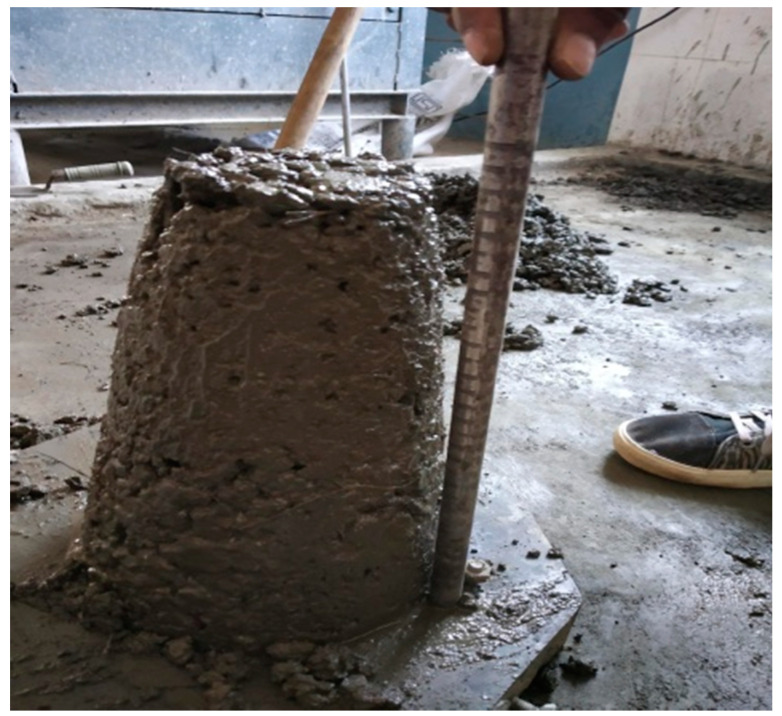
Slump of CS concrete.

The slump cone test is the basic test adopted to examine the fresh concrete property using its consistency. The slump value obtained is used to determine the workability of the fresh mix for further usage. The slump cone test was performed as per IS 1199: 1959. The apparatus consists of a mold with a height of 30 cm and a top and bottom diameter of 10 cm and 20 cm, respectively; along with a tamping rod of 16 mm diameter and 0.6 m long. The mold is filled in four layers by tamping each layer with 25 blows with the rounded end of the tamping rod. In order to measure the slump value, the mold should be cleaned outside and raised immediately after levelling the top surface. After the concrete has subsided, the slump value was measured as the difference of height between the top surface of the mold and the highest point of the specimen. For the shallow condition, the degree of workability was very low and the slump value was minimum. The light reinforced section degree of workability was low and the slump value was between 25 and 75 mm. The heavily reinforced section degree of workability was medium and the slump value was between 75 and 100 mm. The heavily reinforced section without vibration, i.e., Tremie concrete degree of workability was high and slump value was between 100 and 150 mm. The degree of workability of heavy reinforced section was shown in Figure 1.

#### 3.2.2. Compressive Strength

**Figure 2:**
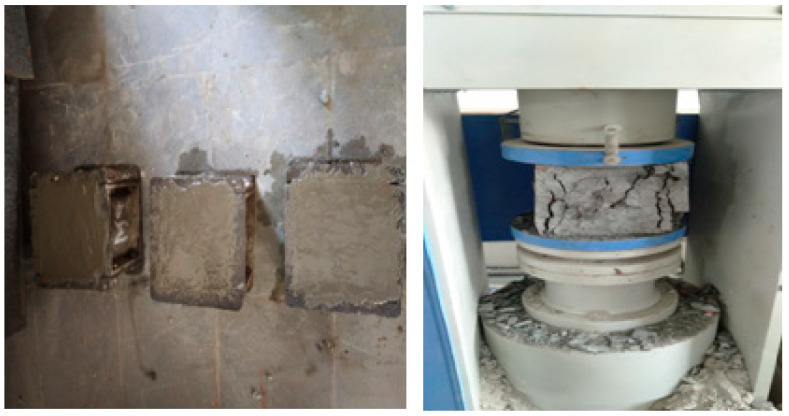
Casting and testing of CS concrete specimen.

Compressive strength is one of the commonly measured hardened concrete properties, used to depict the ability of the material to carry compressive loads without cracking or deflection. Cubes of size 100 mm × 100 mm × 100 mm were cast as per IS 516:1959 to test the compressive strength of concrete. The cast specimens were placed in a compression testing machine of capacity 200 kN and the load was applied gradually (140 kg/cm^2^ per minute) till the failure of the specimen. The load at which the specimen failed was noted as either failure load or ultimate compressive load. The compressive strength of the specimen was calculated by dividing the failure load or ultimate compressive load by its cross-sectional area. For each mix, three cubes were cast and tested, and the average value was taken as the final compressive strength. The test samples of the compressive strength of concrete and plastic failure or load carrying capacity of compressive strength of concrete is shown in Figure 2.

#### 3.2.3. Split Tensile Strength

Although the compressive strength itself is enough to verify the efficiency of the concrete, further confirmations in the opposite directions of responses due to tensile loads should also be checked. Cylindrical specimens of diameter 150 mm and 300 mm length are molded as per IS 516:1959 and subjected to water curing for a period of 28 days. The cylindrical specimens after curing are mounted on the compression testing machine and the load is gradually increased (70 to 140 kg/cm^2^ per minute) until a longitudinal split of the cylinder is observed. The load at which the cylinder splits is taken as the failure load. It is mandatory that at least minimum three specimens should be subjected for every trail and the average strength is taken as the split tensile strength.

#### 3.2.4. Flexural Strength

Flexural strength represents the load bearing capacity of the concrete prism of size 100 mm × 100 mm × 500 mm cast as per IS 516:1959, subjected to bending due to a two-point load (180 kg/cm^2^ per minute min) applied at a spacing of 1/3 of the specimen length. The beams were tested on a standard flexural strength testing machine of minimum capacity 50 kN.

#### 3.2.5. Modulus of Elasticity

The cylinder specimen with a diameter of 150 mm and 300 mm length as per IS 516:1959 is cast and cured for the required number of days. The cylindrical specimen was then mounted on the platform of a compression testing machine, which transmits the compressive load gradually and the deformation being measured by a longitudinal compressometer gauge. The compressive strain was measured at 2/3 length of the specimen from the center. The experiment is repeated for four consecutive cycles, during which the difference in magnitude of deformation almost reaches a constant or less than 5%. The corresponding modulus of elasticity is determined by plotting a graph between the stress and the strain, and the modulus of elasticity was obtained with respect to the secant modulus.

#### 3.2.6. Shrinkage Study

This shrinkage of concrete could be measured using a comparator reading as per ASTM C 157. The length comparator helped to measure the length variation in concrete models. The initial comparator reading will be recorded after 24 h of casting and being kept in a wet condition for 28 days. The model is dried, and then kept in a laboratory with measured humidity for a minimum of 7 days. The comparator reading is recorded at 7, 14, 28, 56, 90, and 180 days. The dried shrinkage is then calculated as the difference in length of the cured specimen and the length when it is completely dried. This test method measures the change in length other than the outwardly applied loads and the test results are obtained in a controlled environment (temperature and moisture).

## 4. Results and Analysis

### 4.1. Results of Concrete with Coconut Shell

The first phase of experimentation provides the experimental findings of concrete with coconut shell as a partial replacement of coarse aggregate. A detailed discussion on the fresh and hardened concrete properties of CS concrete was made in order to find the optimum percentage of replacement.

### 4.2. Fresh Property of CS Concrete

**Figure 3:**
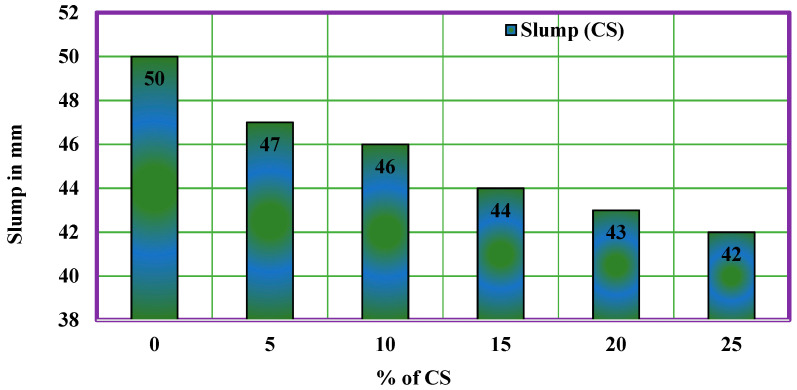
Slump of CS concrete.

The fresh concrete property of the CS concrete is measured using the slump cone test. The conventional concrete shows a slump value of 50 mm, whereas the concrete with 5% of CS shows a decrease in slump value as 47 mm. Further, the slump values of all CS concrete have shown a decrease in slump and the maximum decrease in slump was observed for CS concrete with 25% coconut shell. The slump value obtained for different ratios of CS concrete is provided in Table 2, and Figure 3 shows the variation of slump for various CS mixes. The reason behind the decrease in slump could be due to the high-water absorption of coconut shell.

### 4.3. Compressive Strength of CS Concrete

**Figure 4:**
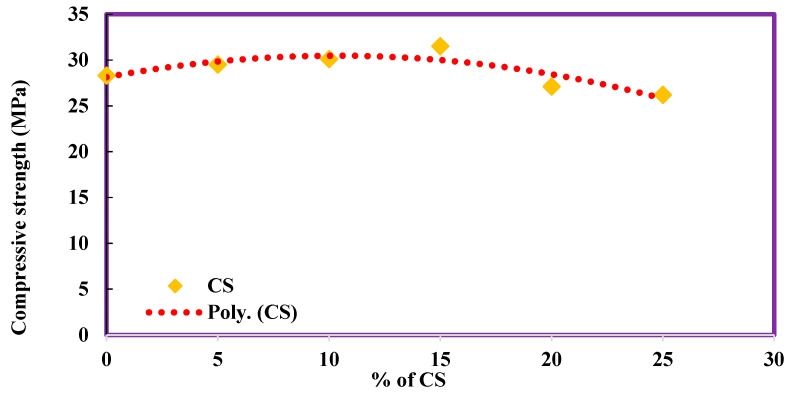
Compressive strength of CS concrete.

The compressive strength of concrete without coconut shell was obtained as 28.3 MPa. With the addition of 5%, 10%, and 15% CS, there is an increase in compressive strength value as 29.5, 30.1, and 31.5, respectively, which is 4.24%, 6.36%, and 11.31% higher than the conventional concrete. The compressive strength value has started decreasing with the further addition of CS by 4.24% and 7.42% for 20% and 25% CS in concrete. The change in variation of compressive strength was plotted as a polynomial function, as shown in Figure 4. The details of compressive strength values are shown in Table 3. The regression equation obtained shows R^2^ value (0.7677), i.e 76.77% accuracy on the developed polynomial equation.

### 4.4. Split Tensile Strength of CS Concrete

The split tensile strength concrete with different percentages of CS was given in Table 4.

**Figure 5:**
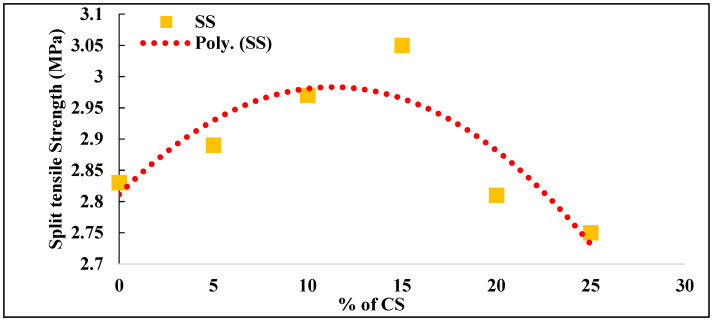
Split tensile strength of CS concrete.

The split tensile strength of concrete without coconut shell was obtained as 2.83 MPa. With the addition of 5%, 10%, and 15% CS, there is an increase in split tensile strength value as 2.89, 2.97, and 3.05, respectively, which is 2.12%, 4.95%, and 7.77% higher than the conventional concrete. The split tensile strength value started decreasing with the further addition of CS by 0.71% and 2.83% for 20% and 25% CS in concrete. The relation between the split tensile strength and percentage of coconut shell was plotted as a polynomial function, as shown in Figure 5. The regression equation obtained shows an R^2^ value of 0.7591, which means the equation can predict split tensile strength of CS concrete with an accuracy of 75.91.

### 4.5. Flexural Strength of CS Concrete

**Figure 6:**
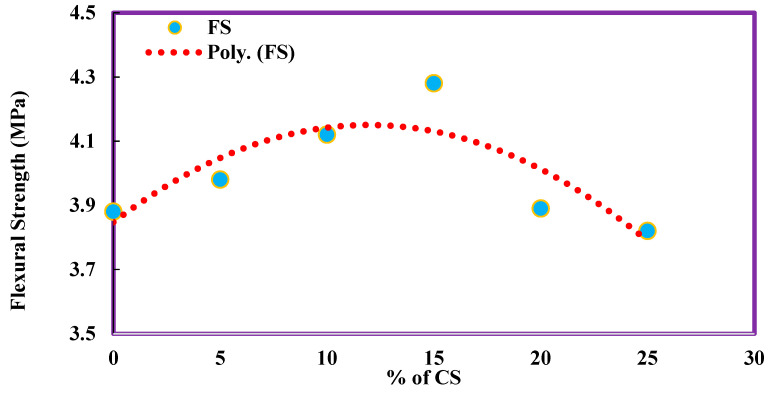
Flexural strength of CS concrete.

The flexural strength of concrete without coconut shell was obtained as 3.88 MPa. With the addition of 5%, 10%, 15%, and 20% CS, there is an increase in flexural strength value as 3.98, 4.12, 4.28, and 3.89, respectively, which is 2.58%, 6.19%, 10.31%, and 0.26% higher than the conventional concrete. The flexural strength value of 25% CS has alone shown a decrease in strength by 1.55%. Flexural strength results have shown a different trend when compared to compressive strength and split tensile strength. The flexural strength concrete with different percentages of CS are given in Table 5. The change in variation of flexural was plotted as a polynomial function, as shown in Figure 6. The regression equation obtained shows the R^2^ value (0.7069), i.e., a 70.69% accuracy on the developed polynomial equation to predict the flexural strength of CS concrete.

### 4.6. Modulus of Elasticity

**Figure 7:**
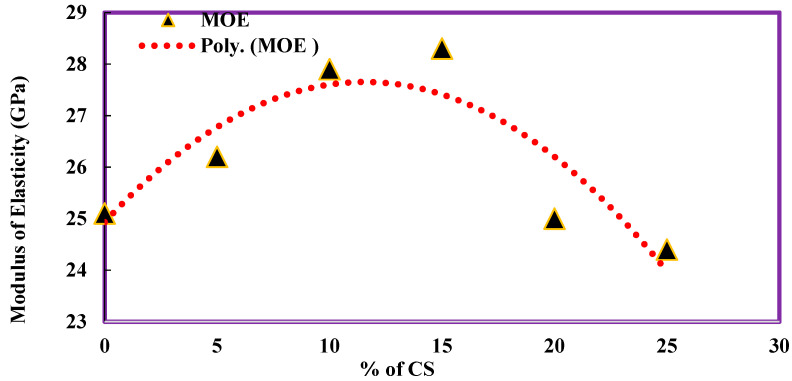
Modulus of elasticity of CS Concrete.

Table 6 shows the modulus of elasticity of concrete with CS as coarse aggregate replacement. The control concrete obtained a value of 25.1 GPa as the modulus of elasticity. There is an increase in the modulus of elasticity by 4.38%, 11.16%, and 12.75% for concrete with 5%, 10%, and 15% of coconut shell, respectively, in concrete. There is a decreasing trend followed in the modulus of elasticity for 20% and 25% CS by 0.4% and 2.79%. The relation between the modulus of elasticity and percentage of coconut shell was plotted as a polynomial function, as shown in Figure 7. The regression equation obtained shows a R^2^ value of 0.7826, which means the equation can predict the split tensile strength of CS concrete with an accuracy of 78.26.

In phase one, the experimental work on the partial replacement of coconut shell with coarse aggregate in concrete was worked out. The results of fresh and hardened properties of concrete were found using a slump cone test, compressive strength, split tensile strength, flexural strength, and modulus of elasticity, and the optimum percentage of replacement for coarse aggregate with CS was found to be 15%. Although the flexural strength decreased for 20% of CS, and as the percentage increase in strength was less, 15% CS concrete was taken as the optimum % replacement following the trend of compressive strength and split tensile strength. The reason behind this is that coconut shell along with the fibers form a layer around the concrete pores, which improves the strength of the concrete. Table 7 shows the polynomial regression equation generated for the relationship between the strength and percentage of replacement of CS.

### 4.7. Workability

**Figure 8:**
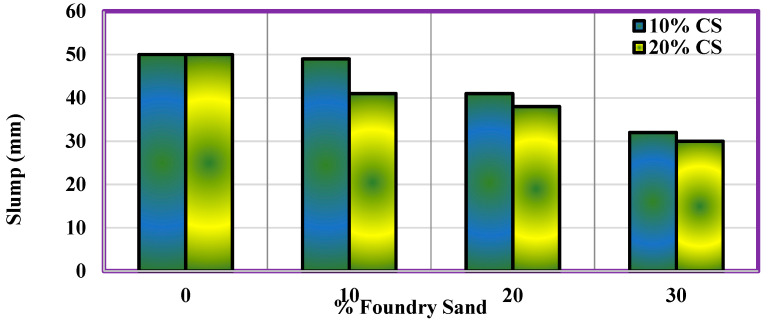
Slump test results.

The concrete mixture workability is calculated through the standard slump cone test, subsequently for the second phase of the experimentation, these outcomes can be entered into the above table. Figure 8 shows the continuous rise in the percentage of WFS and CS, which makes the slump values decrease. The percentage decrease in slump value is more for concrete with a higher percentage of CS (20% CS). A study indicates that the concrete mixture’s ductility relies on its shape, dimensions, grading, and type of aggregates. Although the compensation for a higher water absorption is through the use of a super plasticizer, there still exists a decrease in the workability due to the fine particles of WFS. The chemical properties of waste foundry are mentioned in Table 8.

### 4.8. Compressive Strength

Figure 7 explores the second phase of the experimentation of compressive strength with various ratios of WFS and CS. A total of 10% CS Concrete compressive strength was higher than the 20% CS concrete identified. A total of 10% CS Concrete compressive strength shows an increasing percentage of WFS.

**Figure 9:**
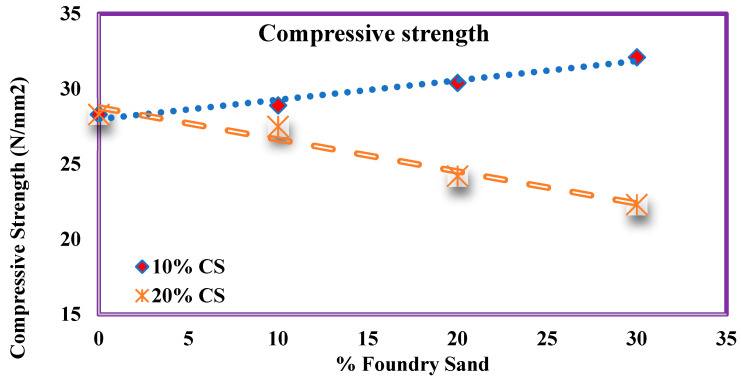
The compressive strength of concrete contains different ratios of FS and CS.

**Figure 10:**
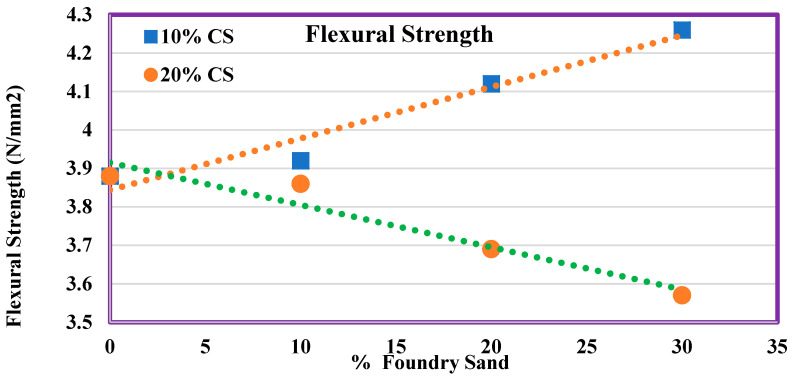
Flexural strength of concrete contains various ratios of FS as well as CS.

**Figure 11:**
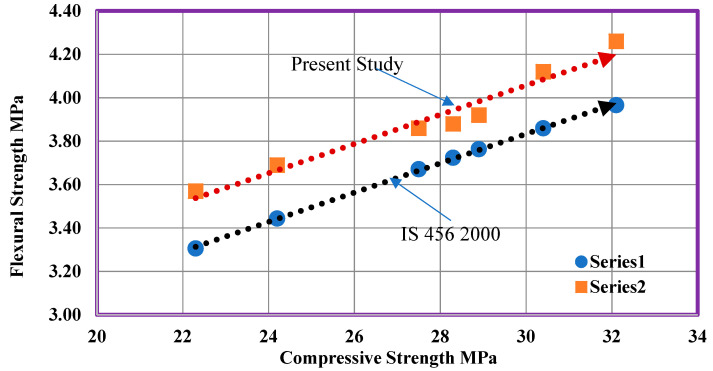
A study of flexural strength and compressive strength of concrete, containing different percentages of FS as well as CS.

**Figure 12:**
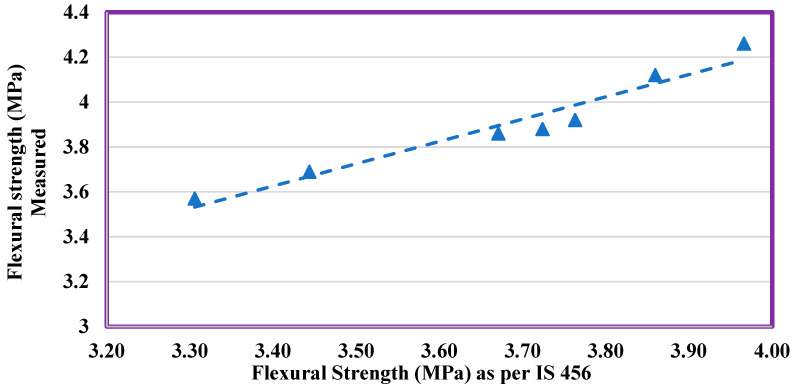
Measured flexural stress vs. computed flexural strength.

**Figure 13:**
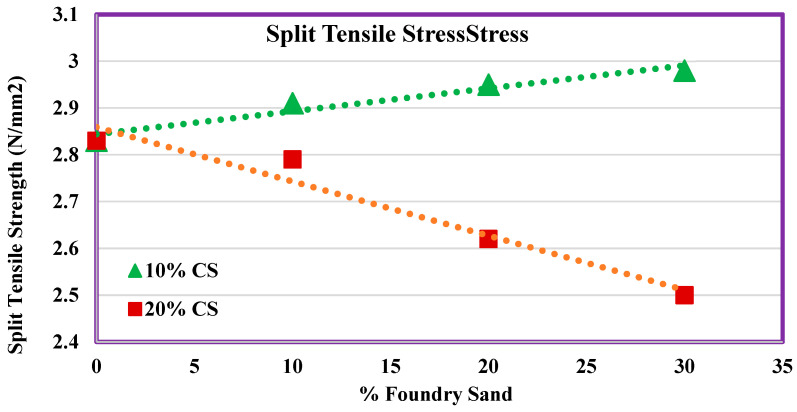
Split tensile strength contains various percentages of FS as well as CS.

A total of 20% CS shows a decreasing trend in strength for all percentages of WFS. Figure 9, Figure 10, Figure 11, Figure 12 and Figure 13 indicate that the same trend was followed for split tensile strength and flexural strength. After 28 days, the normal concrete gained the strength of 28.3 MPa, which was increased by 2.1%, 7.2%, and 13.4% for mixes M11, M12, and M13. However, the strength decreases by 2.8%, 14.5%, and 21% for mixes M21, M22, and M23. Among all mixes, M13 showed more compressive strength than another mixes.

### 4.9. Flexural Strength

Figure 10 shows the second phase of the experimentation of flexural strength, which uses various ratios of WFS and CS. This also shows the same trend of compressive strength results. The concrete conducted a laboratory test and gained a flexural strength of 3.88 MPa at 28 days, which increased by 1.1%, 6.2%, and 9.8% for mixes M11, M12, and M13. However, the flexural strength decreased by 0.5%, 4.9%, and 7.9% for mixes M21, M22, and M23.

M13 exhibits a higher flexural strength when compared to other mixes. Only a marginal decrease in flexural strength was found for concrete made with 20% CS and all the values are less than 10%. A linear relationship in the form of y = ax + b is used for fitting the figures with a correlation coefficient R^2^ value more than 0.9. The high value of R^2^ indicates that there exists a strong relationship between the percentage addition of WFS and CS with that of the strength values.

The compressive strength of concrete calculated for 28 days was analyzed with the flexural strength of concrete. The experimental value of the flexural strength of concrete for 28 days was referred with the values according to IS 456:2000. The values obtained were shown in Table 9. The flexural strength outcomes from the current study are higher than the values as per IS 456:2000. The estimated equation of flexural strength as per IS 456:2000 is given by

Fr = 0.7 √fck (MPa).(1)

Figure 11 shows the comparison of flexural strength and concrete compressive strength, which contains various ratios of WFS as well as CS. Compressive strength has a direct relationship with flexural strength, and it acts as an index for flexural and split tensile strength. The relationship among the strengths is influenced by various factors such as method of testing, type of aggregate, quality of concrete, and admixtures [8,9,10].

Figure 12 indicates that the flexural strength measured using Equation (1) was plotted against the experimental values. There exists a strong relationship with the measured and experimental flexural strength values, which is evident from the R^2^ value (0.94).

### 4.10. Split Tensile Strength

The second phase of the experimentation of split tensile strength contains various percentages of WFS as well as CS, as shown in Figure 6. In a month, the controlled concrete gained the strength of 2.83 MPa, which was increased by 2.8%, 4.2%, and 5.3% for mixes M11, M12, and M13. However, the strength decreases by 1.4%, 7.4%, and 11.6% for mixes M21, M22, and M23. Among all mixes, M13 showed higher split tensile stress than other mixes.

This split tensile strength for 10% CS was more in comparison with 20% CS. Similar to compressive strength and flexural strength, there exists a high correlation coefficient for split tensile strength as well, as shown in Figure 13. There exists a positive correlation for 10% CS whereas there exists a negative correlation for 20% CS.

### 4.11. Drying Shrinkage

The drying shrinkage strains were calculated based on the equation below (2).

**Figure 14:**
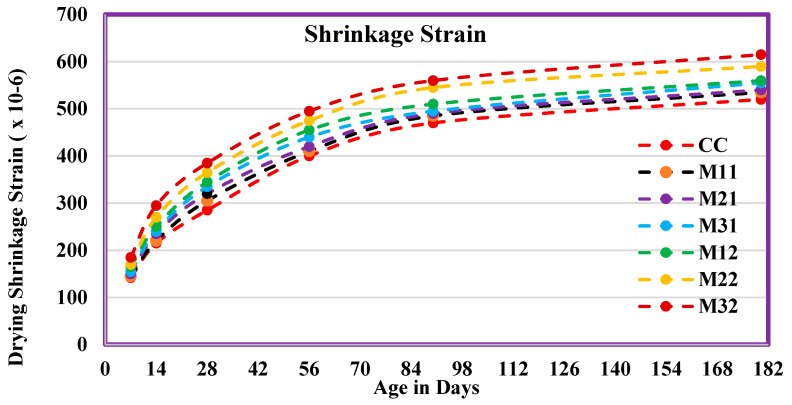
Drying shrinkage strain contains different percentages of FS and CS.

ε_sh_ = (CRi − CR)/L, (2)

where ε_sh_ is the drying shrinkage strain (mm/mm), CRi is the initial comparator value of the specimen, CR is the comparator value, and L is the model’s gauge stretch. Figure 14 shows that the concrete shrinkage strain from drying out contains different percentages of WFS and CS. At the time of extending the age of drying, the shrinkage strain also extends for all mixes. The shrinkage strain was at a higher rate during the initial days of drying and gradually increased at later stages. At the time of extending the percentage of WFS and CS, the drying shrinkage strain also extends to a higher level than normal concrete [11,12,13,14,15,16,17].

The shrinkage strain values of mix M11 were very close to the CC. Similarly, the percentage variation of strain values for mix M21 are between 2.2% and 3.5% for all ages of curing. The higher shrinkage strain values for M11–M32 mixes were attributed to factors such as the compressibility of aggregates, presence of clay, and excessive humidity of WFS and CS aggregates. Nevertheless, all the mixtures’ shrinkage values stayed within the limit of 800 × 100^−6^ mm/mm.

In phase two of the experimental work, the combination of both foundry sand and coconut shell remains a partial replacement for fine aggregate and coarse aggregate undergone in a concrete mix. The replacement of foundry sand, up to the 30%, remains as a filler in forming void free concrete. Improving the durability and the strength of concrete in a mechanical test was the main goal. As far as coconut shell is concerned, we have already shown in phase one that a 15% replacement shows optimum value. In combination of both coconut shell and foundry sand in same concrete, a 25% replacement of foundry sand and 15% replacement of coconut shell remains the optimum value of replacement of aggregates in concrete [18,19,20,21,22].

### 4.12. SEM Analysis

**Figure 15:**
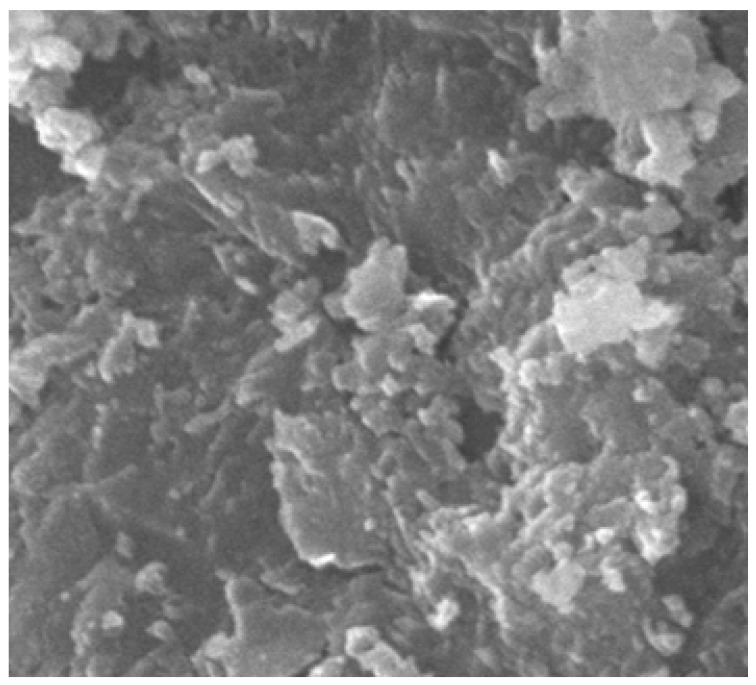
Normal concrete.

**Figure 16:**
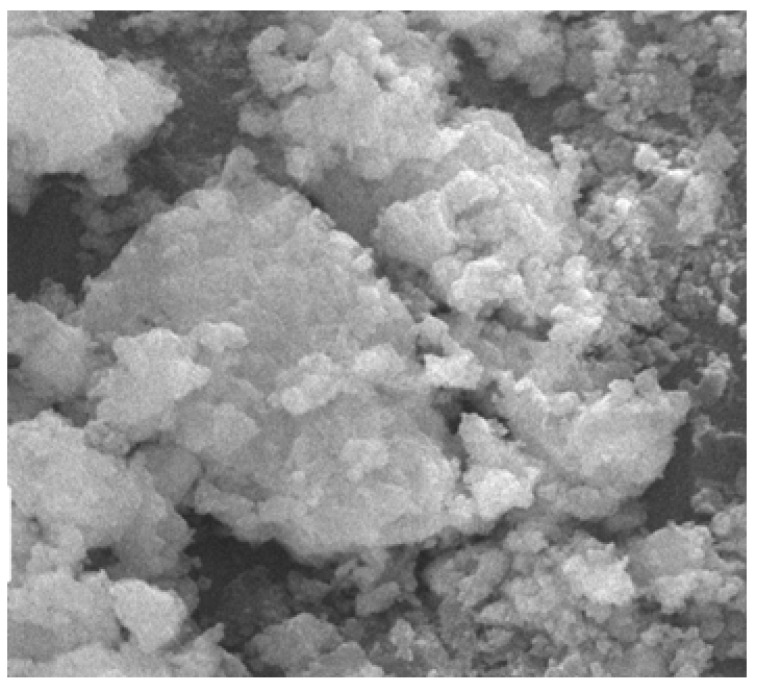
Foundry sand replacement in concrete (30%).

**Figure 17:**
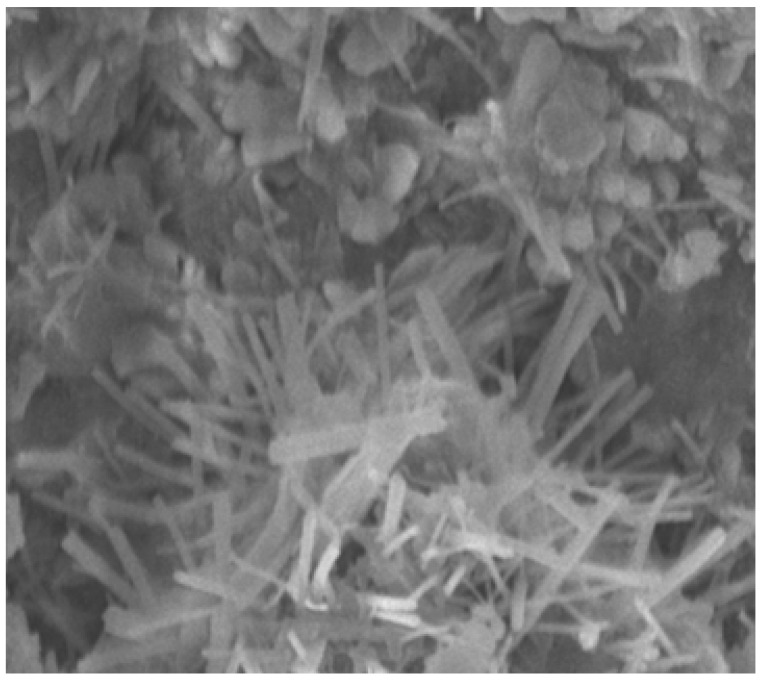
Coconut shell replacement of in concrete (15%).

**Figure 18:**
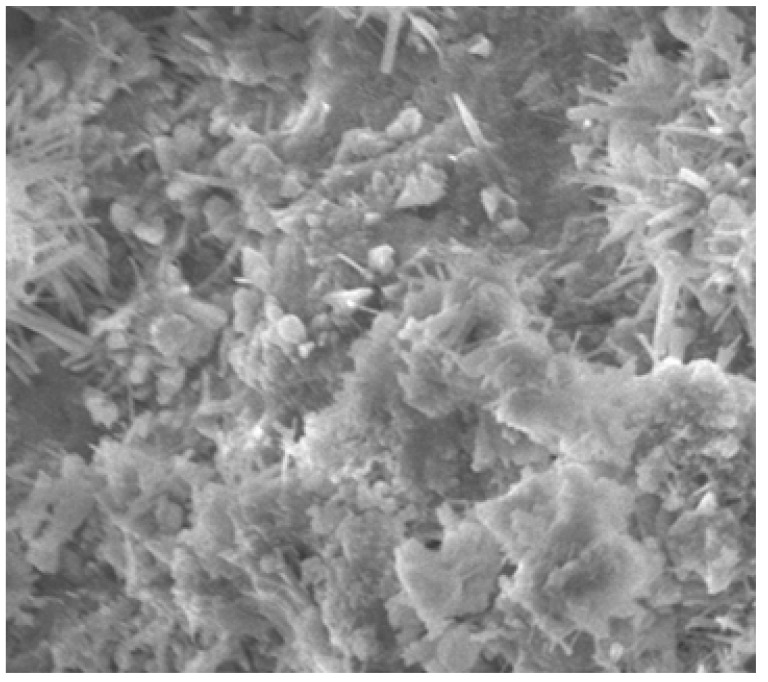
Foundry sand and coconut shell replacement in concrete.

The SEM analysis images are given for four categories. For normal concrete, concrete with coconut shell 15% replacement for coarse aggregate are shown in Figure 15 and Figure 16. Fine aggregate was replaced by 30% by weight using foundry sand and a combination of both coconut shell and foundry sand replacement for aggregates are shown in Figure 17 and Figure 18. Normal concrete shows the formation of CSH gel over the normal aggregates and has a clear surface. Figure 14 depicts the formation of lumps/spherical particles in the case of concrete with 15% CS. The lumps/spherical particles reveal that there is a weak bond between concrete and coconut shell. A needle-like structural formation is found for concrete with 30% FS, as shown in Figure 15. This is due the presence of silica content in foundry sand, and it reacts with the cement to form a dense structure and makes the concrete durable and impermeable. Figure 16 shows the irregular formations of particles in the concrete, which is due to the combination of CS-FS products. These formations fill up the voids present in the concrete and makes it dense and durable. Hence, the increase in the strength of concrete incorporating CS-FS is due to the above-mentioned characteristics. The concrete with a dense microstructure has good mechanical properties, which is validated through various strength results such as compressive strength, split tensile strength, and flexural strength [23,24,25,26].

## 5. Conclusions

In summary, the study concludes the following findings:

1. An increase in the percentage of waste materials in concrete decreases its workability, which is revealed through a slump cone test. The reason behind this could be due to the finer particles of WFS and water absorption of CS;
2. Amalgamation of WFS in concrete increases the strength, whereas the CS inclusion reduces the durability of concrete. Concrete durability is increased by WFS particles, which fills up the void space and makes the concrete denser;
3. The polynomial equations were derived to study the relationship between the percentage of CS and the strength properties. All the strength properties exhibited more than a 70% correlation with the CS%;
4. A linear regression equation was framed to study the different relationship parameters, such as %FS vs. compressive strength, split tensile strength, flexural strength, and also compressive strength vs. flexural strength with high correlation levels (above 90%);
5. The difference between the measured experimental values and the values predicted based on codal equations (IS456) were also studied and compared;
6. At the time of extending wastages in concrete, the drying shrinkage of concrete also increases. Because of excessive humidity of WFS and CS aggregates, the magnitude of drying shrinkage increases. Nevertheless, all the mixtures’ shrinkage values stay within the limit of 800 × 100^−6^ mm/mm;
7. Through the micro-structural characterization, the reason behind the increase in mechanical properties was found. The addition of CS of more than a certain limit leads to a decrease in the strength properties due to the smooth surface and delamination between the layers of concrete. The addition of FS showed better characteristics than CS concrete. Moreover, CS-FS concrete exhibited superior properties over individual replacements due to the formation of a dense matrix.

## Figures and Tables

**Table 1:** Strength properties of WFS-WCS concrete.

Mix	% WFS	% CS	Slump	Compressive Strength (MPa)	Flexural Strength (MPa)	Split Tensile Strength (MPa)
CC	-	-	50	28.3	3.88	2.83
M11	10	10	49	28.9	3.92	2.99
M21	20	10	41	30.4	4.12	2.95
M31	30	10	32	32.1	4.26	2.88
M12	10	20	41	27.5	3.86	2.79
M22	20	20	38	24.2	3.69	2.62
M32	30	20	30	22.3	3.57	2.5

**Table 2:** Slump of CS concrete.

Mix Code	CS %	Slump mm
CC	0	50
CS5	5	47
CS10	10	46
CS15	15	44
CS20	20	43
CS25	25	42

**Table 3:** Compressive strength of CS concrete.

Mix	CS	Compressive Strength	Increase in Compressive Strength
	%	MPa	%
CC	0	28.3	-
CS5	5	29.5	4.24
CS10	10	30.1	6.36
CS15	15	31.5	11.31
CS20	20	27.1	−4.24
CS25	25	26.2	−7.42

**Table 4:** Split tensile strength of CS concrete.

Mix	CS	Split Tensile Strength	Increase in Split Tensile Strength
	%	MPa	%
CC	0	2.83	-
CS5	5	2.89	2.12
CS10	10	2.97	4.95
CS15	15	3.05	7.77
−CS20	20	2.81	−0.71
CS25	25	2.75	−2.83

**Table 5:** Flexural strength of CS concrete.

Mix	CS	Flexural Strength	Increase in Flexural Strength
	%	MPa	%
CC	0	3.88	-
CS5	5	3.98	2.58
CS10	10	4.12	6.19
CS15	15	4.28	10.31
CS20	20	3.89	0.26
CS25	25	3.82	−1.55

**Table 6:** Modulus of elasticity of CS concrete.

Mix	CS	Modulus of Elasticity	Increase in Modulus of Elasticity
	%	GPa	%
CC	0	25.1	-
CS5	5	26.2	4.38
CS10	10	27.9	11.16
CS15	15	28.3	12.75
CS20	20	25	−0.40
CS25	25	24.4	−2.79

**Table 7:** Regression equation for CS concrete.

Relation Ship	Polynomial Equation	Regression Co-Efficient
% of CS vs. Compressive strength	y = −0.0218x^2^ + 0.4515x + 28.132	R^2^ = 0.7677
% of CS vs. split tensile strength	y = −0.0013x^2^ + 0.0304x + 2.8114	R^2^ = 0.7591
% of CS vs. Flexural strength	y = −0.0021x^2^ + 0.0507x + 3.8475	R^2^ = 0.7069
% of CS vs. Modulus of elasticity	y = −0.0204x^2^ + 0.4706x + 24.932	R^2^ = 0.7826

**Table 8:** Chemical properties of foundry sand.

Composition %	FS
Sand and Silica SiO_2_	95.6
Calcium Oxide CaO	0.29
Iron Oxide Fe_2_O_3_	0.36
Alumina Al_2_O_3_	1.71
Magnesium oxide MgO	0.43
Potassium Oxide K_2_O	1.61

**Table 9:** Comparison of experimental and predicted values of flexural strength.

Mix	% WFS	% CS	Compressive Strength (MPa)	Flexural Strength (MPa)	
				Experimental	IS 456:2000
CC	-	-	28.3	3.88	3.72
M11	10	10	28.9	3.92	3.76
M21	20	10	30.4	4.12	3.86
M31	30	10	32.1	4.26	3.97
M12	10	20	27.5	3.86	3.67
M22	20	20	24.2	3.69	3.44
M32	30	20	22.3	3.57	3.31

## Data Availability

Not applicable.

## References

[1] Khatib J.M., Herki B.A., Kenai S. (2013). Capillarity of concrete incorporating waste foundry sand. Constr. Build. Mater..

[2] Jayaprithika A., Sekar S.K. (2016). Stress-strain characteristics and flexuralbehaviour of reinforced Eco-friendly coconut shell con-crete. Constr. Build. Mater..

[3] Kumar V.R.P., Gunasekaran K., Shyamala T. (2019). Characterization studyon coconut shell concrete with partial replacement of cement by GGBS. J. Build. Eng..

[4] Kanojia A., Jain S.K. (2017). Performance of coconut shell as coarse aggregatein concrete. Constr. Build. Materials..

[5] Siddique R., Singh G. (2011). Utilization of waste foundry sand (WFS) in concrete manufacturing. Resour. Conserv. Recycl..

[6] Gunasekaran K., Annadurai R., Chandar S.P., Anandh S. (2017). Study for the relevance of coconut shell aggregateconcrete non-pressure pipe. Ain Shams Eng. J..

[7] Nithya M., Priya A.K., Muthukumaran R., Arunvivek G.K. (2017). Properties of concrete containing waste foundry sand for par-tial replacement of fine aggregate in concrete. Indian J. Eng. Mater. Sci..

[8] Shahidan S., Leman A.S., Senin M.S., Hannan N.I.R.R. (2017). Suitability of Coconut Shell Concrete for Precast CoolWall Panel-A Review. MATEC Web Conf..

[9] Mysore T.H.M., Patil A.Y., Raju G.U., Banapurmath N.R., Bhovi P.M., Afzal A., Alamri S., Saleel C. (2021). Investigation of Me-chanical and Physical Properties of Big Sheep Horn as an Alternative Biomaterial for Structural Applications. Materials.

[10] Akhtar M.N., Khan M., Khan S.A., Afzal A., Subbiah R., Bakar E.A. (2021). Determination of Non-Recrystallization Temperature for Niobium Microalloyed Steel. Materials.

[11] Nagaraja S., Nagegowda K.U., Kumar V.A., Alamri S., Afzal A., Thakur D., Kaladgi A.R., Panchal S., Saleel C.A. (2021). Influ-ence of the Fly Ash Material Inoculants on the Tensile and Impact Characteristics of the Aluminum AA 5083/7.5SiC Compo-sites. Materials.

[12] Sathish T., Kaladgi A.R.R., Mohanavel V., Arul K., Afzal A., Aabid A. (2021). Experimental Investigation of the Friction Stir Weldability of AA8006 with Zirconia Particle Reinforcement and Optimized Process Parameters. Materials.

[13] Sharath B.N., Venkatesh C.V., Afzal A. (2021). Multi Ceramic Particles Inclusion in the Aluminium Matrix and Wear Characteri-zation through Experimental and Response Surface-Artificial Neural Networks. Materials.

[14] Sathish T., Mohanavel V., Ansari K., Saravanan R., Karthick A., Afzal A., Alamri S., Saleel C.A. (2021). Synthesis and Charac-terization of Mechanical Properties and Wire Cut EDM Process Parameters Analysis in AZ61. Materials.

[15] Meignanamoorthy M., Ravichandran M., Mohanavel V., Afzal A., Sathish T., Alamri S., Khan S.A., Saleel C.A. (2021). Micro-structure, Mechanical Properties, and Corrosion Behavior of Boron Carbide Reinforced Aluminum Alloy (Al-Fe-Si-Zn-Cu) Matrix Composites Produced via Powder Metallurgy Route. Materials.

[16] Kumbar S.S., Jadhav D.A., Jarali C.S., Talange D.B., Afzal A., Khan S.A., Asif M., Abdullah M.Z. (2021). Enhancement in Ca-thodic Redox Reactions of Single-Chambered Microbial Fuel Cells with Castor Oil-Emitted Powder as Cathode Material. Materials.

[17] Akhtar M.N., Sathish T., Mohanavel V., Afzal A., Arul K., Ravichandran M., Rahim I.A., Alhady S.S.N., Bakar E.A., Saleh B. (2021). Optimization of Process Parameters in CNC Turning of Aluminum 7075 Alloy Using L27 Array-Based Taguchi Method. Materials.

[18] Kaur G., Siddique R., Rajor A. (2013). Micro-structural and metal leachate analysis of concrete made with fungal treated waste foundry sand. Constr. Build. Mater..

[19] Nithya M., Maheswaran G., Senthil Kumar S. (2016). Experimental investigation on impact of sand mining in coastal regions and potential reuse of silica sand as construction material. Indian J. Geo-Mar. Sci..

[20] Pennarasi G., Soumya S., Gunasekaran K. (2019). Study for the relevance ofcoconut shell aggregate concrete paver blocks. Mater. Today: Proc..

[21] Siddique R., Aggarwal Y., Aggarwal P., Kadri E.H., Bennacer R. (2011). Strength, durability, and micro-structural properties of concrete made with used-foundry sand (UFS). Constr. Build. Mater..

[22] Chandrashekar A., Chaluvaraju B.V., Afzal A., Vinnik D.A., Kaladgi A.R., Alamri S., C. A.S., Tirth V. (2021). Mechanical and Corrosion Studies of Friction Stir Welded Nano Al2O3 Reinforced Al-Mg Matrix Composites: RSM-ANN Modelling Ap-proach. Symmetry.

[23] ASTM C 157 (2008). Standard Test Method for Length Change of Hardened Hydraulic-Cement Mortar and Concrete.

[24] ACI Committee 209 (2005). Report on factors affecting Shrinkage and creep of hardened concrete. Concr. Int..

[25] Etxeberria M., Pacheco C., Meneses J.M., Berridi I. (2010). Properties of concrete using metallurgical industrial by-products as aggregates. Constr. Build. Mater..

[26] IS: 456-2000 (2000). Indian Standard Plain and Reinforced Concrete—Code of Practice.

